# The family talk intervention prevent the feeling of loneliness - a long term follow up after a parents life-threatening illness

**DOI:** 10.1186/s12904-024-01611-3

**Published:** 2024-12-12

**Authors:** Emily Bergersen, Cecilia Olsson, Maria Larsson, Ulrika Kreicbergs, Malin Lövgren

**Affiliations:** 1 Karlstad University, Universitetsgatan 2, Karlstad, 651 88 Sweden; 2 Inland Norway University of Applied Sciences, Box 400, Elverum, 2418 Norway; 3 Lovisenberg Diaconal University, Lovisenberggata 15B, Oslo, 0456 Norway; 4 Great Ormond Street Institute of Child Health, University College London, 30 Guilford Street, London, WC1N 1EH UK; 5 Marie Cederschiöld University, Box 11189, Stockholm, 100 61 Sweden

**Keywords:** Psychosocial support, Family intervention, Children as next-of-kin, Family talk intervention, Family communication

## Abstract

**Background:**

The psychosocial needs of families in which a parent is affected by life-threatening illness and has dependent children are extensive. However, few family-based interventions have been scientifically evaluated and even fewer have been evaluated long term. Therefore, the specific objectives of this study were to describe the parents’ perceptions of the timing and length of FTI in relation to the illness trajectory, to explore what activities learnt by the FTI still were practiced in the long-term and what content of FTI was perceived as most valuable to cope in the long-term.

**Methods:**

This qualitative study involved a follow-up with nine parents (ill parents, *n* = 3, coparents, *n* = 6) 4 to 5 years after participating in FTI, where one parent was cared for in specialised palliative homecare. FTI is a psychosocial family-based intervention that consists of 6–11 manual-based meetings with the families led by an educated interventionist. FTI focuses on facilitating family communication about illness-related subjects, supporting parenting, and making the children’s needs visible. The data collection consisted of interviews and was analysed according to the phenomenographic method, focused on variations in perceptions.

**Results:**

The parents perceived FTI as a way to alleviate feelings of loneliness, and some families were still using the obtained communication tools at the time of the interview. They also perceived that FTI contributed to the children being more open about their own feelings and thoughts. However, the parents wanted extended support after FTI ended based on their individual needs, for example, during and after bereavement, deteriorated health or occasional challenges faced by children in crisis. The parents perceived the peer support gained in conjunction with FTI as important social and emotional support both during and after the intervention. The interventionists were perceived as professional persons who promoted open and honest communication during FTI.

**Conclusion:**

FTI is found to promote family communication both in a short- and long-term perspectives according to parents. They also found FTI useful in reducing their feelings of loneliness. Support over a longer period of time is desired and extra FTI meetings may strengthen the family as a whole in parallel with additional support for parents and children during the illness trajectory and in bereavement. They received support in dealing with strong and difficult emotions and learned conversational techniques that they still used at the time of the interview, indicating that the lessons learnt was integrated and valuable many years after the last FTI meeting.

**Supplementary Information:**

The online version contains supplementary material available at 10.1186/s12904-024-01611-3.

## Background

Communication within the family in general is what binds family members together into a unit, which is done by helping them learn from each other, develop self-confidence, express thoughts and feelings to each other, maintain family rules, present expressions and behaviours and resolve conflicts [1, 2]. However, when a family is affected by life-threatening illness, they are forced to talk about difficult topics and family communication becomes challenging. The challenges become even greater as there are dependent children in the family and the parents have to talk to them about topics such as prognosis and impending death.

Life-threatening illness in parents with dependent children has been shown to be associated with high risks of psychosocial distress for all family members [3, 4]. The ill parents struggle to cope with their illness and treatment while upholding family routines and maintaining parenting roles [5, 6]. Similarly, the coparents strive to assume the roles of both parents while managing their partners’ care and balancing their own needs with the needs of their partner and children [7, 8]. Furthermore, the children go through distressing experiences, such as uncertainty in the parents’ illness progression, changed family life, worry and fear of death and dying [9]. A common concern for both parents and their children is how to communicate about the illness [5, 6]. Parents often find it difficult to talk to their children about death and dying [6, 8, 10]. Previous research has shown that open communication within the family may lead to increased satisfaction related to a well-functioning family life among parents and have positive effects on children’s psychological health [6, 11, 12]. Family communication can be improved by involving the entire family in an intervention because it often enhances family members’ understanding of their own and each other’s perspectives and impacts and consequences of the parent’s illness [2, 13, 14]. This suggests that interventions aiming to strengthen families’ communication strategies might be an appropriate approach. The children are often aware that something is wrong and want honest information, feeling that they are given sparse information about the parent’s illness, but the children do not want to bother their parents with questions and concerns [6, 14–17].

Despite this, systematic reviews have shown that there are few evaluated family interventions for families with dependent children [18–20]. Even fewer family interventions have been evaluated in a long-term perspective. A psychosocial family-based intervention (Family Talk Intervention, FTI) that was originally developed for families in the psychiatry context was pilot-tested among families with dependent children where a parent had a life-threatening illness [21–23]. The main goals of FTI are to facilitate family communication about illness-related subjects, support parenting, and making the children’s psychosocial needs visible, which corresponds with what earlier research has found that these families need. In the evaluations in conjunction with the end of FTI [21–23], our research group found that the parents reported that family communication improved as family members learned communication strategies that facilitated open sharing of thoughts and feelings. A majority of the families found FTI well-structured and adaptable to the needs of each family [21]. The children reported that they felt seen, heard and acknowledged during the intervention period, but also that FTI increased their knowledge about the illness and that they felt it became easier to talk to their parents [14, 24]. However, to date, no long-term evaluation of FTI has been carried out, and we therefore wanted to identify what perceived value the intervention had for the families in the years following participation. Knowledge of how sustainable the effects of FTI are important to explore for further development of the intervention. The specific objectives of this study were to describe the parents’ perceptions of the timing and length of FTI in relation to the illness trajectory, to explore what activities learnt by the FTI still were practiced in the long-term and what content of FTI was perceived as most valuable to cope in the long-term.

## Methods

### Design

This is a qualitative long-term follow-up study with parents participating in FTI during 2017–2018 [21]. A phenomenographic approach was applied.

#### The family talk intervention

FTI is manual-based and is led by FTI-educated interventionists, which, in the present study, included a social worker and deacon. The interventionists were solely responsible for the implementation of the intervention and did not participate in either the interviews or the data analysis. FTI has an eclectic approach that includes psycho-educative, narrative and dialogical ways of working. The psycho-educative element focuses on increased knowledge about the illness and related subjects. The narrative element involves the family’s own stories and is central in FTI; family members share their stories with each other and create a joint family history. The dialogical way of working focuses on making problematic situations visible by making the children’s voices heard, sharing experiences within the family and seeing all the family members’ different perspectives [25]. FTI entails six meetings, with intervals of one to two weeks between. If the intervention is interrupted unexpectedly and cannot be finished as scheduled because of extraordinary circumstances, extra meetings (7–11 in quantity) are available. (Table 1). The meetings in FTI are organised within the same family, which meant that the meetings were not open to other families or other external parties.

**Table 1:** Organisation of FTI meetings implemented in 2017/2018

Meeting No.	Attendees	Meeting agenda
1 and 2	The parent(s)	Their experiences of the situation, as well as the consequences of the diagnosis for each family member. During the meeting, each child’s situation will be discussed, including strengths, problems, worries, the situation in school and with friends, their social network and the child’s knowledge of the disease.
3	The child(ren)	The child’s life situation, feelings, understanding of the disease, hobbies, the relationship with their parent(s) and siblings and the social network. The interventionists identify protective factors from the child’s narrative (e.g., well-functioning school), as well as risk factors (e.g., poor social network).
4	The parent(s)	Focuses on planning the family meeting. The family members narratives are taken into account and serve as a guide for the upcoming family meeting.
5	The family	‘The family talk’. It consists of questions and issues raised earlier by the family members. This family meeting can be seen as a starting point for communication within the family.
6	The family	A follow-up with all family members. The meeting is guided by the family members’ needs, for example, regarding communication and parenting.
7–11		If the intervention is interrupted unexpectedly and cannot be finished as scheduled because of extraordinary circumstances, extra meetings are available.

**Table 2:** Demographic characteristics of participants and their familie

Gender	Man (*n* = 1) Woman (*n* = 8).
Age (min- max)	40–61 (median = 49).
Ethnicity	Families of Swedish ethnicity (*n* = 6) Families of mixed ethnicity (*n* = 2) Families of non-European ethnicity (*n* = 1).
Role within the family	Ill parent (*n* = 3) Coparent (*n* = 6)
Diagnosis of ill parent	Cancer (*n* = 9).
Relationship status	Widow (*n* = 5). Married (*n* = 3). Single (*n* = 1).
Years since parent’s death	5 years (*n* = 5)
Number of children (min-max)	1–4 (median = 2)
Children’s age (min- max)	4–22 (median = 12).
Number of children participating in the interview (min-max)	0–2
Duration of interview (min- max)	16–67 min (median = 30 min)

### Setting and participants

The present qualitative study involved interviews with a purposive sample consisting of nine parents (ill parents *n* = 3, coparents *n* = 6) four to five years after participating in FTI in families in which one parent had a life-threatening illness and was cared for in specialised palliative homecare in Stockholm, Sweden (Table 2). The specialised palliative homecare service provided 24-hour support to patients and their families by multiprofessional teams who had specialist competence in palliative care. In Sweden, a palliative home care team may consist of palliative medicine specialists, specialist nurses, nutritionists, occupational therapists, psychologists, social workers, physiotherapists, and priests [26, 27]. Eligible families for FTI were those that included a parent with a life-threatening illness and who was enrolled in specialised palliative homecare service from March 2017 to February 2018. In line with the World Health Organization (WHO), we have referred to life-threatening illness as the parent’s diagnosis most likely to result in death within the foreseeable future, but without giving a specific time frame [28]. The families needed to be able to speak and write Swedish and have at least one child aged between 6 and 19 years, at the time for the intervention.

Out of 20 families that were included in FTI [21] 12 families participated in a survey one year after they had participated in FTI (2017–2018). At the end of the survey, we asked the parents if we could contact them in the future. Of the 12 families, 10 consented to be contacted for eventual additional data collection. These 10 families were contacted by telephone (by MLö, UK) during the spring 2022, with an invite to participate in an interview study about FTI. Nine participants consented. The reason for not participating (*n* = 1) was lack of time. The families themselves decided which family members would participate in the interviews. In all the families, one of the parents (ill or healthy) chose to take part. In five of the families, the ill parent had died. In the remaining four families, three families consisted of two parents (participating in the study: ill parents *n* = 2, coparents *n* = 1), and one family consisted of an ill single parent. All but one of the interviewed parents were women. All participating ill parents had a cancer diagnosis, and all coparents lived/have lived with a partner with cancer. The families had between one and four children (median 2), and the parents’ ages ranged between 40 and 61 years (median 49 years).

### Data collection

The interviews were carried out between March and May 2022 in a place chosen by the families (families home *n* = 4, digitally *n* = 5) and were primarily conducted with the parents. However, the children were physically present in three of the interviews. Some of these children contributed by confirming what the parents expressed during the interviews. A semi structured interview guide was used, and initially, the parents were encouraged to openly describe their memories and perceptions of participating in FTI. The interview guide was structured in such a way that each topic corresponded to a specific objective. Thereafter, in line with the objectives, the parents were asked about their perceptions of the timing of the intervention and what they may have applied in the years since. In addition, the parents were encouraged to share their perceptions of what else might have been needed in FTI and how they ideally wanted to be supported. The length of the interviews ranged from 16 to 67 min (median = 30 min).

### Data management

Before the interview, the families gave informed consent, both verbally and in writing. Regional Ethical Review Board in Stockholm and the Swedish Ethical Review Authority (Dnr. 2017/7–31/1 and 2021–05295) approved the study.

The primary material in the research project, that is, the interviews, was processed only by the research group and stored in a secure cloud service at the university. Thereafter, in line with Swedish and European regulations, the data will be archived at the university and destroyed after 10 years [29].

### Data analysis

Phenomenography was chosen as the method of analysis in order to uncover the qualitatively different ways the parents perceived FTI in the long term (cf. 29–32). Through the phenomenographic analysis, similarities and differences in perceptions were discovered both on an individual and collective level, and clusters of common meanings defined the descriptive categories. The findings are presented in an outcome space, which covers the different descriptive categories and their logical relationship [30]. There are various ways to conduct a phenomenographic analysis [31–34], which is done with accompanying analysis steps. In the present study, the analysis was carried out in seven steps, as inspired by Hyrkäs et al. [35]. In line with the phenomenographic method, emphasis was placed on the second order perspective, that is, *how* the parents perceived FTI [30].

Step 1 involved repeatedly reading transcripts and listening to voice recordings of the entire material to obtain an overall picture. Step 2 consisted of selecting the comments of interest for the research aim, and step 3 consisted of comparing the comments of interest with the content of the transcripts to ensure a proper understanding. Steps 1 to 3 were initially conducted by the first author. The comments and preliminary understanding were then discussed in the research group. Step 4 consisted of the formation of pools of meanings by grouping the comments of the previous stage. In step 5, the similarities and differences of the pool of meanings, naming the emerging categories, and further testing them, by comparing them with the entire data (code-recode). Step 6 involved the generation of descriptions of categories and their interrelationships, that is, if they were hierarchically, horizontally or vertically linked. During steps 4–6, the research group compared and critically examined pools of meanings, categories and outcome space until a consensus had been obtained, using quotations to make the decision trail visible [36]. Finally, step 7 consisted of renaming the categories using concepts that describe the material as well as possible. The analysis process consisted of going back and forth in the different stages of analysis, and all stages of the analysis and outcome space were discussed until a consensus had been reached in the research group.

## Results

The analysis revealed an outcome space consisting of one main category, ‘A way to alleviate feelings of loneliness’, and three descriptive categories consisting of parents’ qualitatively different perceptions of participating in FTI, all described on a collective level. The descriptive categories were as follows: [1] support from professionals and peers [2], strategies to manage strong emotions and difficult issues, and [3] Supporting children to manage the situation. In this context, the term ‘support’ is directly linked to which aspects the participants in the study perceived as supportive in FTI.

The main category summarises the findings at a higher level of abstraction and could be further understood by the descriptive categories. ‘Strategies to manage strong emotions and difficult feelings’ and ‘Supporting children to manage the situation` were mutually influencing, but superior to ‘support from professionals and peers’. The latter descriptive category, contains descriptions of perceptions of FTI as a phenomenon, and is a prerequisite for the remaining two descriptive categories.

**Fig. 1:**
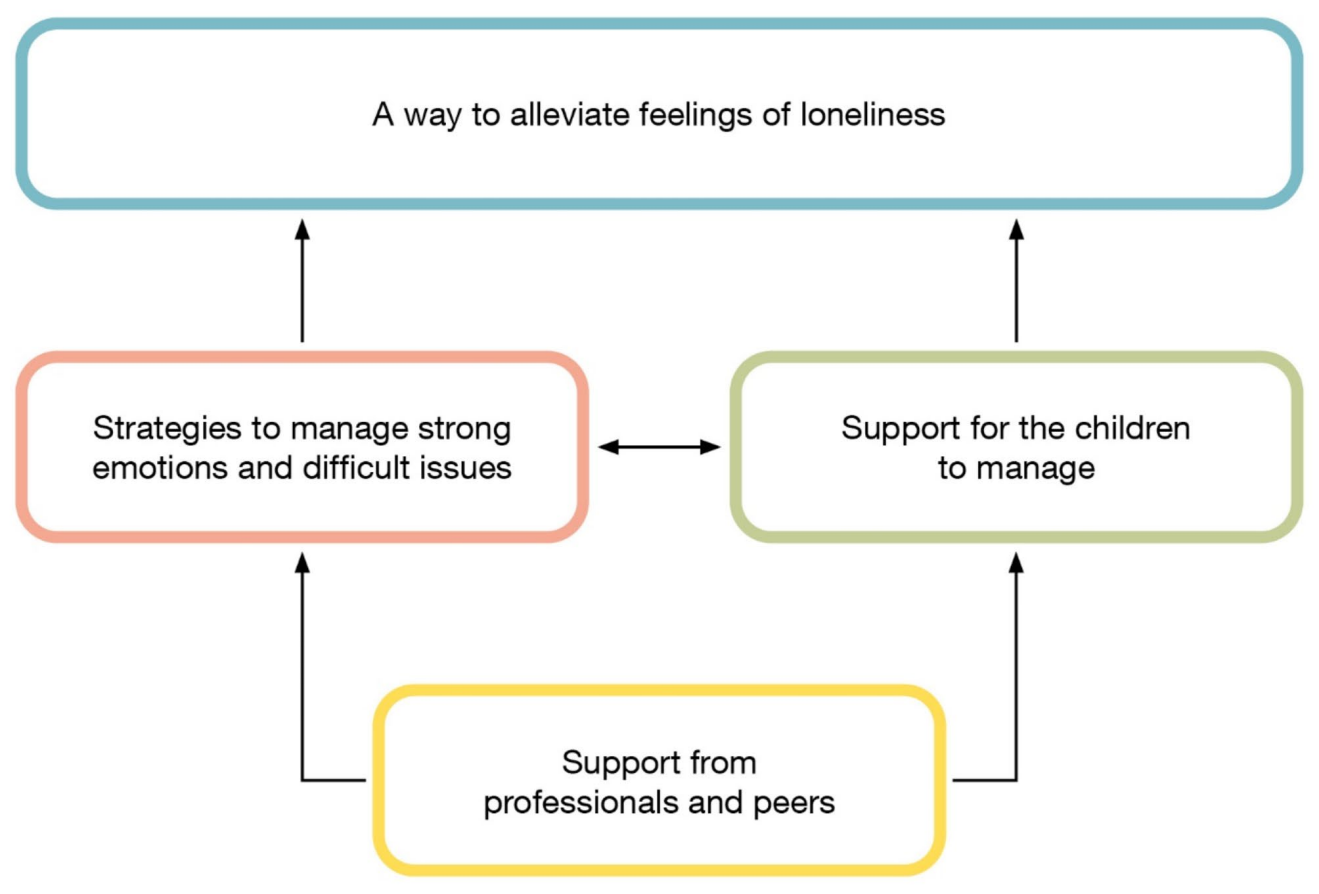
Illustration of the main category, the descriptive categories and their relationships

The Outcome space can emerge differently in various studies and settings, in terms of various hierarchies [34]. The outcome space i.e. the relation between the main category and descriptive categories in the current study, is illustrated in Fig. 1. The figure is organised hierarchically, starting from the descriptive category “support from professionals and peers” and moving upwards to the main category “A way to alleviate feelings of loneliness”.

### A way to alleviate feelings of loneliness

The main category is made up of perceptions that participation in FTI is a way to alleviate feelings of loneliness. At the same time, the parents perceived that if this was to be achieved, the structure and timing of the intervention needs to be adapted to the families’ individual needs. Parents’ perceptions of the timing of FTI varied both according to individual preferences and according to where the ill parent was in the course of the illness at the time of the intervention. Some perceived it as beneficial to participate while the ill parent was still alive and, thus, able to share thoughts and feelings with the rest of the family; the parents perceived that the intervention contributed to greater openness within the family. However, the ill parent’s participation required that the parent still was healthy enough to participate. In cases where families were not able to share their thoughts and feelings because of cognitive limitations that made this impossible, the coparents perceived that the intervention came too late. Greater openness within the family seemed to prevent loneliness, whereas a lack of openness appeared to increase the level of perceived loneliness.

Parents who perceived it as positive that the intervention came early in the course of the illness described that it reduced feelings of chaos and loneliness. Some perceived a relief from the structure FTI provided because it supported decision-making. Others expressed a desire that FTI should be a mandatory intervention for families in difficult situations because it is easier to attend meetings than to initiate support services on one’s own. On the other hand, there were families who perceived that FTI came too early because the chaos they were in was initially overwhelming, making them unresponsive to support provided by the intervention. These families were in greater need of other forms of support, such as crisis management. Support by means of FTI provided when the family is receptive prevents loneliness, while FTI to not mentally ready and/or motivated families is counterproductive.

In addition to the timing of FTI, parents stressed the need for more support after the FTI had ended. The majority of parents perceived that FTI should consist of a greater number of meetings and that the meetings should take place over a longer period of time, that is, over the course of illness and also after the death of those family members left behind. They suggest that the extended support should be adapted to the individuals’ needs and different phases of the illness trajectory, that is, perceptions of content related to practical issues such as adapting the home to the needs of the ill parent, information and preparation of the children for the death of the parent, financial issues such as sickness benefits and wills and bereavement support. The perception of loneliness was strengthened when support meetings through FTI ended and the parents felt left alone with unmet needs.

### Support from professionals and peers

This descriptive category was a prerequisite for the main category and consists of varied perceptions regarding the meaningful traits of the professional interventionists, FTI as an intervention programme in itself and new friendships acquired in conjunction with FTI.

Through FTI, families were able to meet FTI-trained interventionists. A common perception among both ill and coparents was that it was positive to meet someone outside the family who was a professional with specific knowledge in palliative care.

. *It was nice to be able to share thoughts with other people who understood the situation and did not back down. They were not afraid to get into this conversation.* ….

Participant number; four.

There were variations in what parents perceived as meaningful in the professional support they received through FTI. Some emphasised that professionals with a genuine understanding of the situation were important, as well the certainty that they in their role and competence dared to raise sensitive issues. Others emphasised that FTI was a part of a research project, which contributed to the parents’ perceptions of the intervention as a promising form of support. Previously demonstrated promising effects through scientific testing gave the parents faith that the intervention could also help them in their situation.

*… For those who are sceptical*,* you can say that this is based on science. The intervention is based on research and follows a pattern*,* and it has proven to be helpful … I think that is an important argument to emphasise. …*.

Participant number; seven.

FTI interventionists mainly facilitated family communication, but also some practical help (e.g., organising meetings with the school, assisting with the procurement of aids, help with filling in applications, etc.). This was perceived as positive by both the ill and coparents.

The interventionists contributed to the possibility of meeting others in the same situation as themselves by facilitating contact with other families. The parents perceived FTI as positive, and their new network was used in different ways: discussions about difficult feelings and emotional support, practical help and people to socialise with for pleasant events.

. *Yes*,* we have a difficult loss in common. But it’s not like we always sit and cry … we have fun when we meet*,* we are friends and we have a good time together. …*.

Participant number; two.

For most parents, the friendships created during their participation in FTI were still ongoing today, years after the intervention was over. Just knowing that there were others in the same situation as themselves, whether they saw them or not, was perceived as helpful and made some parents feel less alone.

### Strategies to manage strong emotions and difficult issues

This descriptive category was built on the perceptions of how FTI facilitates strategies to manage strong emotions and difficult issues through family communication. The perceptions varied in the parents’ attitude prior to the intervention, involvement of the children and the continuation of family meetings post intervention.

There were variations among parents’ perceptions of both participating and family communication before FTI started. Some perceived FTI as ‘a gift from above’ and that the interventionists had come to relieve them of everyday challenges. Some coparents were hesitant at the beginning of the intervention but had positive experiences both during and afterwards. They were worried that challenging topics could be difficult to manage because, before FTI, there had been challenges within families to have these dialogues, and these perceptions were independent of the family situation. However, participating in FTI was perceived by the parents as giving strategies to manage strong emotions and difficult situations within the family, and some still perceived the participation as meaningful as they continued using the learned strategies. Several coparents perceived that FTI brought family members closer together, both during and after FTI. They perceived that these conversations on sensitive issues would probably not have taken place without the given strategies:

*… you got into these difficult things … what would happen*,* are you afraid of dying? … and really difficult things like this. And we couldn’t talk about that with each other at all! …*.

Participant number; eight.

The challenging dialogues consisted of fear of death, dying and grief, difficult emotions such as anger, anxiety and depression combined with the physical expression of difficult emotions. The strategies obtained through FTI were perceived as particularly valuable in families where, for example, difficult emotions were rarely talked about:

*… It’s especially important for families [us] who come from other cultures*,* to get that support and that help because it’s not as common to talk about … especially about one’s feelings and anxiety and depression and stuff like that. …*.

Participant number; three.

FTI was perceived as valuable support for the families, both by the participating ill parents and by the coparents because they were all in a difficult situation, which had previously made it difficult to bring these sensitive issues up with their children. At the same time, there were parents, both ill and coparents, who perceived that the children were reluctant to engage in this dialogue, which made it difficult for their parents to support them. This was experienced both during and after the intervention, and some thought it was related to the children’s individual needs. The parents perceived that each child had individual needs and that this did not necessarily come out clearly via FTI because of the few meeting times. Most parents perceived that they needed more help in how to support their children and that more meetings would help them meet their children in a more appropriate way. There was also uneasiness among the parents in relation to the future development of mental illness in the children, which came several years after the parent had passed away. Worrying about the children’s psychological development was perceived as an extra burden to carry in an already challenging everyday life for most parents. The parents wished that the interventionists had followed up with each individual child over time. In addition, they also wanted to be supported in their parenthood during difficult events such as bereavement: ‘… *I think you can do this*,* your children seem to need this …*’ Participant number; one.

There were variations in the parents’ perceptions of how they maintained the family meeting structure that FTI also included after FTI had ended. Some families structured their daily lives to allow more time for family meetings and communication since participation in FTI. This was perceived as particularly important among coparents, who, after death, managed everyday challenges on their own. On the other hand, after the death of the ill parent, there were those who found it challenging to do things together as a family, including family meetings, because of changed family dynamics. The deceased parent had left a space that was difficult to fill, and family activities were no longer perceived in the same way. When the ill parent was alive, several of the families also received additional support (e.g., contact with psychologist, counsellor and support groups) after FTI, and they were then able to handle the family meetings. After the parent’s death, the support disappeared, and it became more challenging to maintain the family meetings again.

### Supporting children to manage the situation

This descriptive category is built from the parents’ perceptions regarding the children’s experiences of FTI, individual versus group meetings and unmet needs in the children. The most frequently mentioned motivation for participation in FTI was the parents’ wish for more support for the children.

There were variations in the parents’ perceptions of what benefit the children had from FTI, both during and after the intervention. In some families, the parents perceived that their children had received sufficient support during the intervention, as evidenced by their children being calmer and more confident in the situation afterwards.

Some coparents perceived that FTI was of the greatest value to the child, while other parents, both ill and coparents, perceived that support was insufficient for the children after FTI. These parents were still worried that the children did not speak about their feelings, which was perceived as a risk to their mental well-being.

*… My son really needs to go talk to someone! He reacts in exactly the way you understand he really should; he reacts with anger … And I hope that he takes that opportunity to get help sometime instead of him possibly crashing. …*.

Participant number; five.

There were variations in perceptions regarding whether individual or group meetings with peers were the best solution for the children’s support needs. Some parents perceived that it was positive that the children had separate meetings during FTI because that gave opportunities to speak openly about their own feelings. Both ill parents and coparents described how FTI had made the children talk about things that worried them, thus giving the family an opportunity to deal with the children’s concerns related to sleeping alone, nightmares, fear of the ill parent hurting themselves and perceptions of the parents being angry. Concerns were not always known by the parents.

*… I do not think individual conversations are always the best for the children. They should be given the opportunity to join groups and meet others and share each other’s experiences and grief in some way. …*.

Participant number; nine.

There were also variations in the parents’ perceptions of what kind of support their children needed. Some perceived that their children wanted support to lead a normal life and that they should receive more support regarding that during FTI. Others perceived that the children should get early help from professionals and should be told that all emotions, regardless of what they may be, are normal.

## Discussion

The participants perceived FTI to be both meaningful and useful when it came to facilitating family communication both during and after the intervention. In addition, they found meaning and usefulness in FTI’s support for their children and for their own professional and peer support. Through the various ways in which the families found support from FTI, it contributed to the parents perceiving less loneliness. Loneliness is a phenomenon known in studies done both on life-threateningly ill patients and their caregivers and is a serious condition that can develop into negative health challenges if it is not addressed [37–39]. Within death and dying, three different conditions of loneliness are referred to: social, emotional and existential loneliness [38]. None of the participants in the current study described perceptions that characterised existential loneliness, therefore the focus of the further discussion will be on social and emotional loneliness. However, it is important to mention existential loneliness, as both social and emotional loneliness can develop into existential If not recognized by health care services.

Social loneliness refers to a general experience of sadness and lack of a supportive and encouraging network [38]. Emotional loneliness is characterised by not being able to express one’s feelings and missing someone to feel a genuine connection and belonging with [38]. Existential loneliness is characterised by an unsustainable perception of loneliness that is difficult to handle, even with assistance and support from others. Existential loneliness can appear in two different ways: to perceive oneself as totally disconnected from the world and other people or to experience emotional aspects such as isolation, alienation, emptiness or abandonment [37, 38]. To prevent development of existential and other kinds of loneliness, it is crucial to find support measures that are perceived as useful and meaningful to the families.

For families to perceive FTI as providing optimal usefulness and meaning, the findings in the current study have indicated that timing for the individual family is important. In a palliative care setting, flexibility is a key factor. Participation in an intervention may be affected by the symptom burden in the ill parent and by overwhelmingness because of the caregiver burdens in the coparents [40, 41]. Interventions in palliative care should use adaptive designs, allowing for changes in intensity and duration and focusing on the needs of patients and family members at given times [42]. The participants in the current study perceived a need for more continuity in FTI and that there should have been more meeting times in general. Most prominent was the widows’ needs for more follow-up, even after their partner had died. The desire for more continuity and more meeting times was also highlighted during earlier evaluations of FTI, where the intervention was evaluated after a shorter period of time [21, 24]. The experience that formal support disappears immediately after a family member dies is also supported by other studies [43, 44]. A recent study [45] highlights the challenges the bereaved may face and what service providers must focus on to achieve a successful outcome. Service providers must deal with administrative and financial issues, maintaining parental roles, enduring the memories of the circumstances of the spouse’s/partner’s death, reorganising daily life, facing the loneliness of widowhood and expressing the effects of bereavement.

When the ill parent’s health started to deteriorate, both parents in the current study perceived a form of social loneliness. The most prominent were challenges in talking together within the family. This is also supported by other studies, which have shown that family communication can be demanding when the family is affected by a life-threatening illness. A lack of family communication also makes it difficult to give each other emotional support [46–48]. A recent review study [49] also shows that both the healthy parent and children have a tendency to suppress their own feelings to avoid burdening each other. FTI helped facilitate this through structured family meetings, with specific topics for each meeting that helped to promote talking about difficult issues within the family. In the current study, the structure and the content of FTI was highlighted as a positive factor. The same result was found in an earlier study, where the participants evaluated the intervention one year after completion [21]. This indicates that the structure of FTI itself can have a long-term effect on families. FTI differs from other interventions by being one of the few manual-based interventions within its context [50]. In a new study [51], the need for effective educational interventions when a dying parent has dependent children is highlighted. On a general basis, the overall structure of the way palliative care is organised has been highlighted as an area of improvement in a relatively new review study [52].

In the final phase of the intervention, the parents reported that they experienced an increased sense of security for the future, for both the family and children [23]. In the current study, there were some who still held this view, but there were also several who perceived an uneasiness about the children’s development. Some parents perceived that the children needed to talk more with professionals to avoid developing negative health challenges. This has been supported in a recent review study [49] that points out that open communication with the children will help them cope with the situation in a better way. However, previous studies have also shown that some children prefer distraction and retreating from the illness [53, 54]. The participants in the current study also had different perceptions of the support the children had received from participating in FTI. The fact that FTI could benefit from implementing a child-centred approach for all children to be active participants has previously been highlighted [55]. Children as bereaved individuals grieve in a different way than adults, due to varying degrees of cognitive maturity and the ability to process difficult emotions. After losing a parent, the children are exposed to unique emotional and behavioural challenges that require follow-up from well-trained healthcare professionals. Well-trained healthcare professionals should have the ability to communicate about death in a child-friendly and appropriate manner, recognise and normalise children’s grief reactions, as well as support the bereaved parent in managing children’s grief reactions [56].

The participants in the current study perceived professional and peer support as important. The interventionists’ professional background and the fact that they had a duty of confidentiality meant that the parents perceived that they were met with genuine understanding and were given the opportunity to be completely honest. This was something they also pointed out in the one-year evaluation of the FTI [21]. In a new study [57] carried out both with families and staff within palliative care, the staff’s role is also highlighted; the study highlights the importance of the right professional competence and experience, along with the individual carer’s ability to accept death and talk openly with both patients and relatives about this.

Several parents in current study perceived that their social networks began to distance themselves when an life-threatening illness struck the family. By being put in contact with families in similar situations, the parents perceived that they had someone to share both joys and sorrows with, but above all, someone who really understood what they were going through. This could be considered a measure to prevent both social and emotional loneliness. In a relatively recent review study [58], peer support is also highlighted as a potentially effective measure to achieve an increased quality of life within palliative care. The same results have been found in peer mentorship in another study [59]. On a general basis, families who are in a palliative care situation often prefer support from informal caregivers, such as family and friends, rather than formal caregivers [43].

### Strengths and limitations of the study

All of the present article’s authors have a nursing background, the majority of whom also have clinical expertise within palliative care, so we were conscious of ensuring that the participants’ perceptions were correctly reproduced, which helped us to keep our preconceptions at a distance (credibility) [36]. Credibility was also maintained by having several researchers discussing how we understood the perceptions that emerged from the interviews. By having a conscious relationship with the phenomenon that is FTI, it became possible to take a step back and attempt to put one’s preconceptions aside to the greatest extent possible [30, 31]. Credibility was also ensured by the fact that the participants were interviewed by researchers whom they knew from before and felt confident in, which provided rich data.

Auditability was sought by asking open-ended questions that promoted reflection. The interviewers asked follow-up questions to achieve clarity, which came in addition to the participants being asked to provide concrete examples [60]. During the interviews, the participants were also given follow-up questions and a summary at the end of the interview.

Fittingness was ensured by allowing the participants to choose where they wanted to conduct the interview. This contributed to them being in a safe atmosphere and, thus, better equipped to give reflected and varied input. The parents who took part in the present study formed a varied group in terms of ethnic background, socioeconomic status and family composition, hence resulting in a range of informants representative of the group of parents focused on [60], except for gender, as only one male participated. Greater variation in the participants’ gender would probably have resulted in a broader gender perspective.

In some of the interviews, the children were also present, which may have affected the participants’ ability to be completely honest in their statements through withholding or moderation of responses. However, the children’s presence can also be considered a strength because the parents received confirmation that their statements agreed with the children’s perceptions. In addition, only half of the families asked agreed to participate in the study, so one must take into account that these may be participants who were most positively disposed of FTI in the first place which may have resulted in an overstated and favoured impression of the intervention. If more families had agreed to participate, we could have gained perceptions related to existential loneliness, which we did not see in the current participants. The number of participants was relatively small, but within phenomenography, as with other qualitative methods, the focus is on the in-depth knowledge of the interviews rather than on a large number of participants [36]. Some of the interviews were also relatively short in duration. Despite the limited length of some of the interviews, we still experienced a form of richness because not much new information appeared in the interviews we conducted. In addition, phenomenography emphasises that the number of participants cannot be so large that it becomes challenging to handle the collected data [36]. Despite this, the respondents gave comprehensive answers that contributed to rich data material.

## Conclusion

The parents perceived FTI as meaningful and useful in relation to support in dealing with strong and difficult emotions and learned conversational techniques, both during the intervention and at the long-term follow-up, which was four to five years after participation, indicating that the lessons learnt was integrated and valuable many years after the intervention. Some parents perceived that FTI was useful for the children and their ability to talk openly about feelings and thoughts, but there was still more room for individual adaptations and more follow-up after the end of the intervention. Parents perceived the interventionists as genuine and professional persons with whom they could speak openly and honestly. Peer support that was organised by the interventionists under FTI was perceived as valuable by the parents, and several benefited from this social network.

The combination of the various measures implemented during FTI led to several parents experiencing an alleviated feeling of loneliness with the prerequisites that the intervention is timed according to the family’s needs and life situation, individualised adaptations and continuity for both parents and children.

To date, just over 50 people have completed their FTI training with focus on families with palliative care needs and are thus qualified to lead FTI. The FTI-education involving families with palliative care needs where dependent children are involved is available at Marie Cederschiöld University in Stockholm, Sweden. FTI is also running as full-scale studies now, effectiveness-implementation hybrid design, in several different contexts, but also as bereavement support [61]. FTI is also now being trialled in a digital format to increase accessibility and efficiency for both interventionists and participants.

## Electronic supplementary material

Below is the link to the electronic supplementary material.

Supplementary Material 1

## Data Availability

The datasets generated and/or analysed during the current study are not publicly available because individual privacy could be compromised but are available from the corresponding author on reasonable request.

## Abbreviations

FTI: Family talk intervention
