# Supplementary material for: The family talk intervention prevent the feeling of loneliness - a long term follow up after a parents life-threatening illness
Source: BMC Palliat Care. 2024 Dec 12;23:281. doi: 10.1186/s12904-024-01611-3 (PMC11639116; doi:10.1186/s12904-024-01611-3)
Supplement: Supplementary file 1 — Supplementary Material 1 [file 12904_2024_1611_MOESM1_ESM.pdf]

Interview guide, long-term follow-up of the Family Talk Intervention (FTI)

• Could you please tell us about your participation in the support program, FTI?

To refresh their memory, ask them to tell about the support needs they had as a family during their participation in FTI and what support they received when they participated in the FTI. This applies to them as a family and any individual family member.

With the help of the field notes, help the family to remember e.g., give their goals with the participation in FTI.

• Did the support program come at the right time for you? Too late? Or too early?

• Was there anything in the support program (FTI) that you have benefited from since then? (the family as a whole, any individual family members)

Depending on the response of the above question, ask further - of the support you describe you received, have you continued to use it? If not, how come?

- What type of support did you want/missed out of from the time from diagnosis and onwards?

  What support do you think was missing in the support program (FTI)?

- What kind of support have you received since the end of the support program (FTI)? (the family as a whole, any individual family members)

  If they have unmet needs, have they supported each other or have they received professional support? If so, by whom and in what way (school, employer)?

  If bereaved: Is there any type of support, you missed not having since the death of your loved one? What support have you received?

- Do you have any suggestions for how support to families like yours, could look like? (the family as a whole, any individual family members
